# The Important Role of Perituberal Tissue in Epileptic Patients with Tuberous Sclerosis Complex by the Transcriptome Analysis

**DOI:** 10.1155/2020/4980609

**Published:** 2020-10-15

**Authors:** Shuqiang Li, Huijie Shao, Liansheng Chang

**Affiliations:** ^1^Department of Urology, The First Affiliated Hospital of Zhengzhou University, No. 1 Jianshe East Road, Zhengzhou, Henan 450052, China; ^2^Department of Neuro Intensive Care Unit, The First Affiliated Hospital of Zhengzhou University, No. 1 Jianshe East Road, Zhengzhou, Henan 450052, China

## Abstract

Epilepsy is most common in patients with tuberous sclerosis complex (TSC). However, in addition to the challenging treatment, the pathogenesis of epilepsy is still controversial. To determine the transcriptome characteristics of perituberal tissue (PT) and clarify its role in the pathogenesis of epilepsy, GSE16969 was downloaded from the GEO database for further study by comprehensive bioinformatics analysis. Identification of differentially expressed genes (DEGs), functional enrichment analysis, construction of protein-protein interaction (PPI) network, and selection of Hub genes were performed using R language, Metascape, STRING, and Cytoscape, respectively. Comparing with cortical tuber (CT), 220 DEGs, including 95 upregulated and 125 downregulated genes, were identified in PT and mainly enriched in collagen-containing extracellular matrix and positive regulation of receptor-mediated endocytosis, as well as the pathways of ECM-receptor interaction and neuroactive ligand-receptor interaction. As for normal cortex (NC), 1549 DEGs, including 30 upregulated and 1519 downregulated genes, were identified and mainly enriched in presynapse, dendrite and axon, and also the pathways of dopaminergic synapse and oxytocin signaling pathway. In the PPI network, 4 hub modules were found between PT and CT, and top 5 hub modules were selected between PT and NC. C3, APLNR, ANXA2, CD44, CLU, CP, MCHR2, HTR1E, CTSG, APP, and GNG2 were identified as Hub genes, of which, C3, CD44, ANXA2, HTR1E, and APP were identified as Hub-BottleNeck genes. In conclusion, PT has the unique characteristics different from CT and NC in transcriptome and makes us further understand its importance in the TSC-associated epilepsy.

## 1. Introduction

Tuberous sclerosis complex (TSC), an autosomal dominant inherited disease caused by TSC1 or TSC2 mutation, affects approximately 2,000,000 individuals worldwide and has the typical clinical manifestations of hamartomas in multiple organs throughout the body, including the brain, skin, kidney, lung, eye, and heart [1]. Especially, the disorders of epilepsy, intellectual disability, autism, and other structural lesions occurred in the brain are the leading cause of morbidity and mortality for TSC patients [2]. Of which, epilepsy is most common, accounting for 70-90% [2]. Even though great progresses have been made in recent years, management of epilepsy remains most challenging and controversial [3]. Therefore, it is very urgent to further clarify the pathogenesis of epilepsy.

Benefiting from the advanced development of molecular biology, it has been found that the overactivation of mTOR pathway caused by TSC mutation plays a key role in the development of cerebral cortical lesions [4]. Consequently, mTOR inhibitors have been proven effective in reducing the frequency of epilepsy in TSC patients besides antiepileptics [5]. However, in practice, there are still a considerable number of TSC patients finally treated by tuber resection due to the refractory epilepsy with poor drug control. Unfortunately, the seizure freedom is harvested by only 71% patients at 1-year follow-up and dropped to 51% at 10-year follow-up after the tuberectomy [6].

The intracranial electroencephalogram (EEG) and histopathological abnormalities found in perituberal tissue (PT) suggested that PT also plays an important role in the pathogenesis of epilepsy in addition to cortical tuber (CT) [7, 8]. It has also been confirmed that patients undergoing tuberectomy with a larger region will get a longer duration of seizure freedom [9]. Furthermore, transcriptional differences among CT, PT, and normal cortex (NC) were also reported by Aronica et al. [10]. However, they mainly focused on the differentially expressed genes (DEGs) between CT and NC and just a brief description of differences between PT and NC, leaving the understanding of transcriptional characteristics of PT still unclear. Therefore, the microarray data were further analyzed in the present study to clarify the gene expression features of PT and explore the role of PT in the pathogenesis of epilepsy.

## 2. Data and Methods

### 2.1. Data Collection and Preprocessing

The gene expression dataset GSE16969 was downloaded from the Gene Expression Omnibus (GEO) database (https://www.ncbi.nlm.nih.gov/geo), including 4 CT, 2 PT, and 4 NC samples. All the samples were selected for further analysis in this study. The R language packages “Affy” and “affyPLM” were used for the MicroArray Quality Control (MAQC) and background correcting and normalizing of the raw data, as well as the data merge, probes annotation, and log2 transformation. Subsequently, the missing value was supplemented by the R language package “impute.”

### 2.2. Identification of DEGs

The R language package “limma” was used to screen the DEGs of PT compared with CT and NC. To control the false discovery rate, an adjusted *P* value was calculated by the “Benjamini and Hochberg (BH)” method. The cutoff criteria were adjusted *P* value < 0.05 and ∣log_2_ fold change (FC) | >1. Then, the heat map and volcano plot were performed to achieve the visualization of DEGs by using the package “pheatmap” and “ggplot2”.

### 2.3. Functional Enrichment Analysis of DEGs

The online web tool “Metascape” (http://metascape.org) was utilized to analyze and visualize functional profiles of DEGs. For the enrichment analysis of Gene Ontology (GO) term and Kyoto Encyclopedia of Genes and Genomes (KEGG) pathway, a cutoff value of *P* < 0.01 was considered statistically significant. Furtherly, GO terms and KEGG pathways with a minimum gene count of 3 and an enrichment factor > 1.5 were collected into clusters, which were performed according to the similarity metric calculated by Kappa scores. For terms within a cluster, a term with the minimum *P* value was selected as the representative.

### 2.4. Construction of Protein-Protein Interaction (PPI) Network

The website of Search Tool for the Retrieval of Interacting Genes Database (STRING) (https://www.string-db.org) was used to assess the PPI information with a minimum combined score of 0.4. The plug-in MCODE was used to find hub modules in the whole PPI network, in accordance with the criteria of Degree Cutoff = 2, Node Score Cutoff = 0.2, K − Core = 2, and Max.Depth = 100. Enrichment analysis of GO term and KEGG pathway was furtherly performed for the genes clustered in modules.

### 2.5. Identification of Hub and Hub-BottleNeck Genes

The Cytoscape 3.7.2 was used to further explore the PPI network. The plug-in cytoHubba was used to select the top 10 nodes ranked by Degree, MCC, and BottleNeck, respectively. Genes distributed in the overlapping part of Degree and MCC were identified as Hub genes, and genes distributed in the overlapping part of Degree, MCC, and BottleNeck were identified as Hub-BottleNeck genes. Venn diagram was drawn to reflect the distribution of genes.

## 3. Results

### 3.1. Identification of DEGs

**Figure 1:**
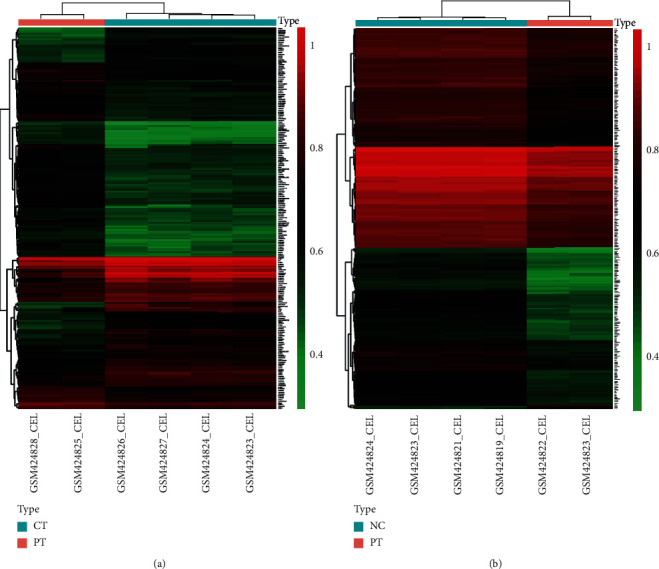
The cluster heat map of DEGs. The red color stands for upregulated genes, and the green color stands for downregulated genes. Black color indicates non-DEGs. (a) Heat map of DEGs between PT and CT; (b) heat map of top 300 DEGs between PT and NC. DEGs: differentially expressed genes; PT: perituberal tissue; CT: cortical tuber; NC: normal cortex.

**Figure 2:**
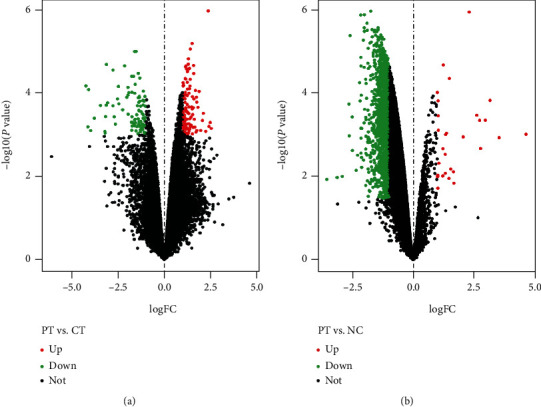
Volcano plot of microarray data. The red and green dots represent the upregulated and downregulated genes, respectively. The black dots represent non-DEGs. (a) Volcano plot of gene distribution between PT and CT; (b) volcano plot of gene distribution between PT and NC. DEGs: differentially expressed genes; PT: perituberal tissue; CT: cortical tuber; NC: normal cortex.

All the 4 CT, 2 PT, and 4 NC samples from GSE16969 were selected for further analysis in this study. The boxplot of normalized unscaled standard errors (NUSE) (Figure S1) and RNA degradation map (Figure S2) was performed for MAQC. According to the criteria of adjusted *P* < 0.05 and ∣logFC | >1, 220 DEGs were identified between PT and CT, including 95 upregulated and 125 downregulated genes shown in Figure 1(a) and Figure 2(a). Concurrently, 1549 DEGs were identified between PT and NC, including 30 upregulated and 1519 downregulated genes shown in Figure 1(b) (top 300) and Figure 2(b).

### 3.2. Functional Enrichment Analysis

**Figure 3:**
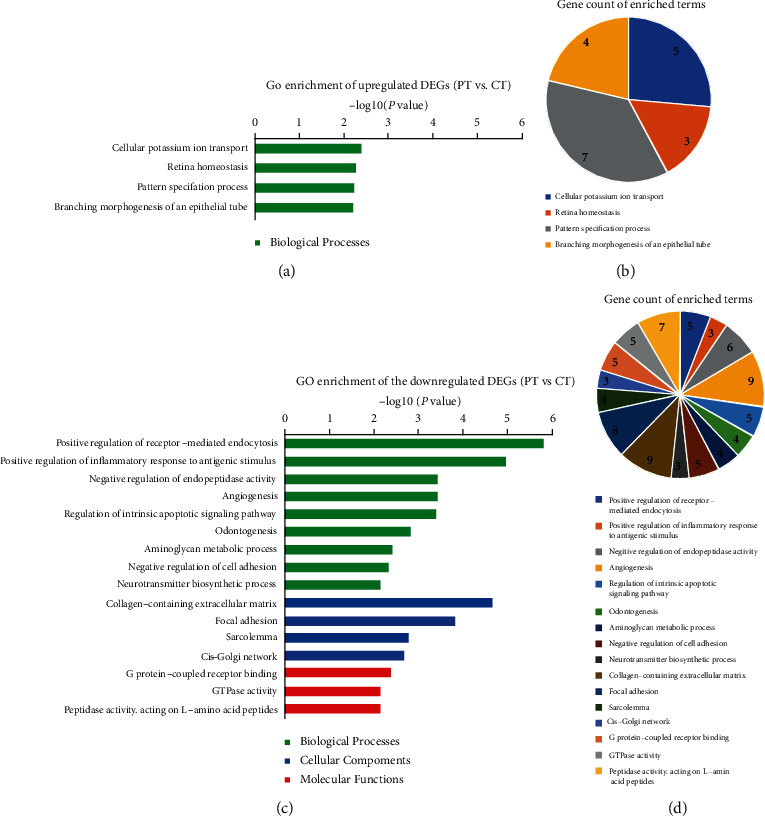
GO enrichment analysis of DEGs between PT and CT. The terms are shown by *P* value and gene count, respectively. (a, b) GO terms of the upregulated DEGs; (c, d) GO terms of the downregulated DEGs. GO: Gene Ontology; DEGs: differentially expressed genes; PT: perituberal tissue; CT: cortical tuber.

**Figure 4:**
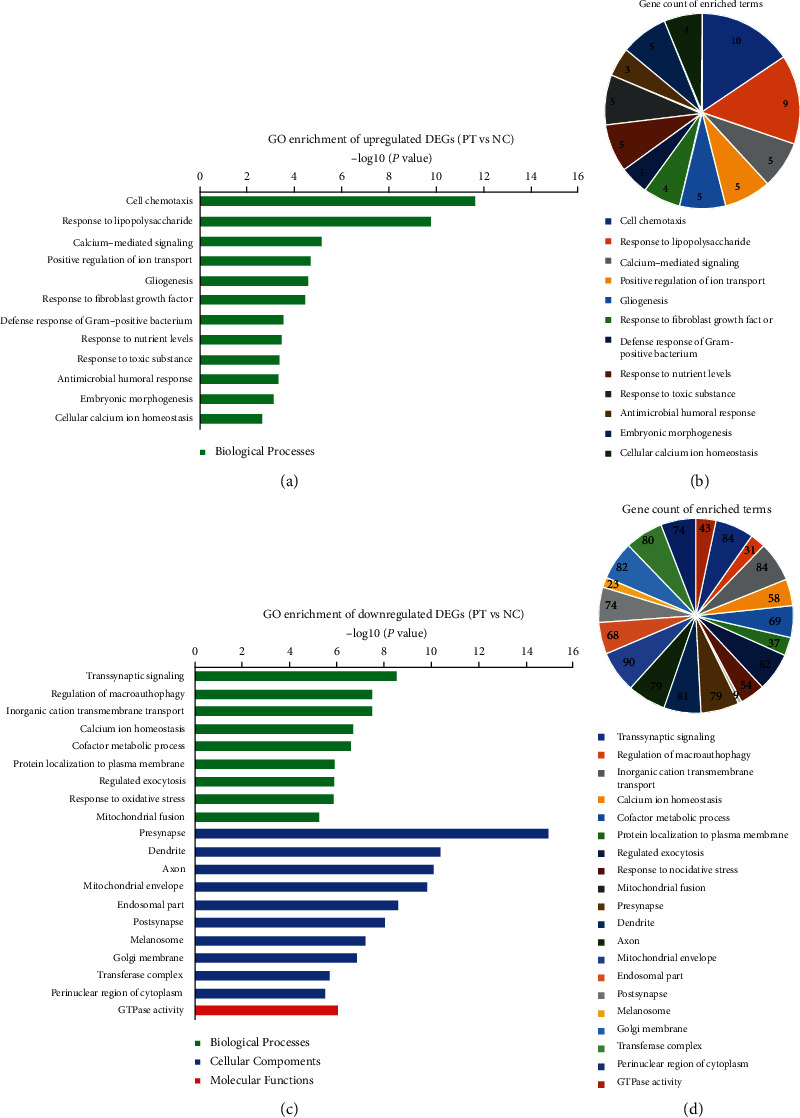
GO enrichment analysis of DEGs between PT and NC. The terms are shown by *P* value and gene count, respectively. (a, b) GO terms of the upregulated DEGs; (c, d) Top 20 GO terms of the downregulated DEGs. GO: Gene Ontology; DEGs: differentially expressed genes; PT: perituberal tissue; NC: normal cortex.

**Figure 5:**
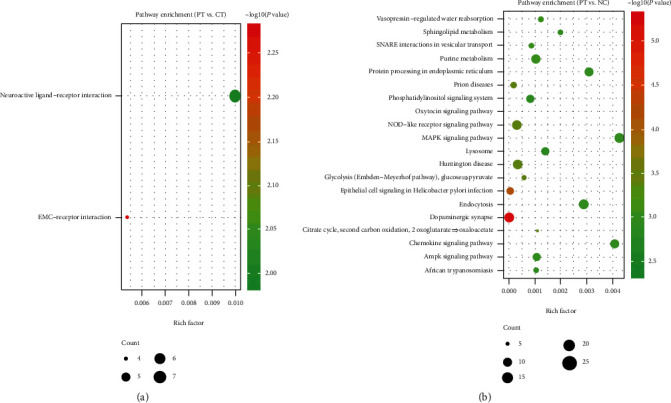
KEGG pathway enrichment analysis of DEGs. (a) KEGG pathways of the DEGs between PT and CT; (b) KEGG pathways of the DEGs between PT and NC. KEGG: Kyoto Encyclopedia of Genes and Genomes; DEGs: differentially expressed genes; PT: perituberal tissue; CT: cortical tuber; NC: normal cortex.

GO and KEGG enrichment analyses were performed by the web tool “Metascape.” In the GO analysis of 220 DEGs between PT and CT, 211 DEGs were recognized and mainly enriched in collagen-containing extracellular matrix, positive regulation of receptor-mediated endocytosis, positive regulation of inflammatory response to antigenic stimulus, and negative regulation of endopeptidase activity, angiogenesis, and focal adhesion (Figure 3). For the DEGs between PT and NC, 1519 out of 1549 DEGs were recognized and mainly enriched in presynapse, dendrite, axon, mitochondrial envelope, transsynaptic signaling, and endosomal part (Figure 4) (top 20, the top 100 GO terms are shown in Figure S5). In the KEGG pathway analysis, the 211 DEGs were mainly enriched in ECM-receptor interaction and neuroactive ligand-receptor interaction, and the 1519 DEGs were mainly enriched in dopaminergic synapse, oxytocin signaling pathway, epithelial cell signaling in helicobacter pylori infection, prion diseases, NOD-like receptor signaling pathway, and Huntington's disease (Figure 5).

### 3.3. Construction of PPI Network and Cluster Analysis

**Figure 6:**
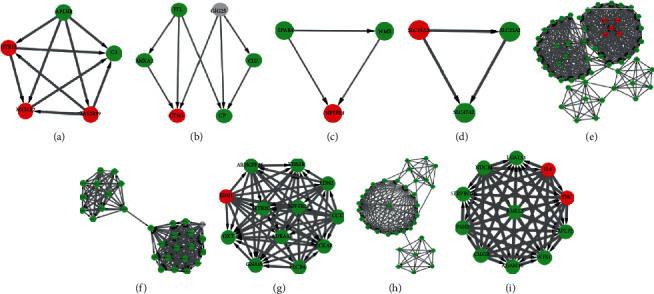
Hub modules of the PPI network. The upregulated genes are marked red, and the downregulated genes are marked green. A non-DEG in (b) is marked gray. (a–d) Modules rank 1-4 in the PPI network of DEGs between PT and CT; (e–i) Modules rank 1-5 in the PPI network of DEGs between PT and NC. PPI: protein-protein interaction; DEGs: differentially expressed genes; PT: perituberal tissue; CT: cortical tuber; NC: normal cortex.

The PPI network of DEGs between PT and CT was constructed via the STRING website, containing 179 nodes and 108 edges (Figure S3). By using the plug-in MCODE, 4 clusters were found as the hub modules in the PPI network finally (Figures 6(a)–6(d)). The genes clustered in hub modules were mainly enriched in azurophil granule lumen and neuroactive ligand-receptor interaction (Table 1). The PPI network of DEGs between PT and NC contained 1411 nodes and 7533 edges (Figure S4). The top 5 clusters were selected as hub modules from the whole PPI network (Figures 6(e)–6(i)). The genes clustered in hub modules were mainly enriched in posttranslational protein modification, mitochondrial translational elongation, and transcription-coupled nucleotide-excision repair (Table 2).

### 3.4. Identification of Hub and Hub-BottleNeck Genes

**Figure 7:**
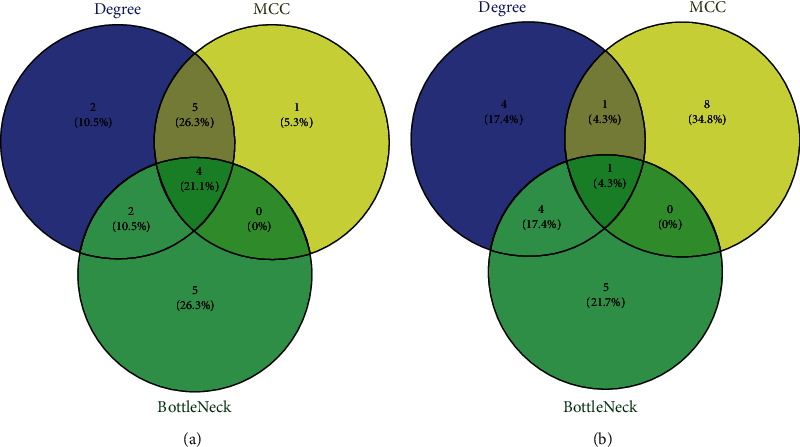
Analysis of the top 10 genes ranked by Degree, MCC, and BottleNeck in Venn diagram, respectively. Hub genes are the ones in overlap of Degree and MCC, and Hub-BottleNeck genes are the ones in overlap of Degree, MCC, and BottleNeck. (a) 5 Hub genes and 4 Hub-BottleNeck genes between PT and CT; (b) 1 Hub gene and 1 Hub-BottleNeck gene between PT and NC. MCC: Maximal Clique Centrality; PT: perituberal tissue; CT: cortical tuber; NC: normal cortex.

The top 10 genes ranked by Degree, MCC, and BottleNeck were selected, respectively, from the PPI network by using the plug-in cytoHubba. Nodes with the same score as the tenth node were also included (Table S1 and S2). According to the Venn analysis, 9 DEGs in Figure 7(a), including 6 upregulated genes (C3, APLNR, ANXA2, CD44, CLU, CP) and 3 downregulated genes (MCHR2, HTR1E, CTSG), and 2 downregulated DEGs (APP and GNG2) in Figure 7(b) distributed in the intersection of Degree and MCC were identified as Hub genes. Furtherly, 5 DEGs (C3, CD44, ANXA2, HTR1E, APP) in the intersection of Degree, MCC, and BottleNeck were identified as Hub-BottleNeck genes (Figures 7(a) and 7(b)).

## 4. Discussion

More than 90% of TSC patients have structural lesions in the central nervous system, which have not only seriously affected their quality of life but also imposed a heavy economic burden on family and society [11, 12]. Even though antiepileptic drugs, mTOR inhibitors, and surgical treatment can be chosen if necessary, the management of refractory epilepsy is still very intractable, attributing to the unclear etiopathogenesis [3, 13, 14]. In addition to CT, which has been confirmed to be able to cause epilepsy, recent literatures have thrown new light on PT, which may also be responsible for epileptogenesis [15].

It has been confirmed that PT plays an important role in the seizure of TSC patients. Although PT is a normal-appearing tissue in magnetic resonance imaging, it has a diffusive connectivity with the epileptogenic CT. PT can not only transmit the abnormal electrical signals from epileptogenic tubers but also cause the intrinsic epileptogenicity [8, 16]. Furthermore, Sosunov et al. have found “microtubers,” which are formed by giant cells and astrocytes together, in PT samples commonly, indicating that PT is a source of seizures in TSC patients [17]. Compared with tuberectomy, surgical removal of CT and PT can better control the recurrence of epilepsy. However, it is not easy to locate epileptogenic tubers accurately in clinic, and it is even more difficult to divide the borders of PT exactly. Therefore, the extent of surgical resection of PT is still controversial and needs further study [3]. Here, we analyzed the different transcriptomic characteristics of PT comparing with CT and NC in detail, aiming to further deepen the understanding of PT and to restate the necessity of surgical resection.

In the previous report, 2501 DEGs had been identified between CT and NC [10], suggesting a huge transcriptome difference. Additionally, we identified 1549 DEGs between PT and NC in the present study, as well as 220 DEGs between PT and CT. Therefore, it further suggests that PT also has its own unique transcriptome characteristics and is quite different from NC and CT, which are also confirmed by the results of hierarchical cluster analysis.

Comparing with CT, 125 upregulated DEGs are found in PT and mainly enriched in the Biological Processes of cellular potassium ion transport and pattern specification process, which are involved in the structure development of neurons and cerebral cortex regionalization [18, 19], as well as the occurrence of epilepsy [20]. Moreover, the 95 downregulated DEGs in PT are mainly enriched in the Biological Processes of positive regulation of receptor-mediated endocytosis and positive regulation of inflammatory response to antigenic stimulus, and also the Cellular Components of collagen-containing extracellular matrix and focal adhesion, which may indicate the decreased perisynaptic extracellular matrix and reduction of cellular hypersensitivity in PT [21, 22], and is also consistent with the result that the expression of inflammation-related microRNAs was increased in CT comparing with PT [23]. The downregulated changes in pathways of neuroactive ligand-receptor interaction and ECM-receptor interaction may also support the secondary role of PT in abnormal transmission of neural electrical signals and occurrence of epilepsy comparing with CT. Hub modules in the PPI network have further illustrated the results.

Based on timely updated databases and better cutoff criteria, more DEGs between PT and NC were identified in the present study than before [10]. The 30 upregulated DEGs are mainly enriched in the Biological Processes of cell chemotaxis, response to lipopolysaccharide, and calcium-mediated signaling, as well as pathways of TNF signaling pathway, cytokine-cytokine receptor interaction, and NF-kappa B signaling pathway, which may aggravate the stability of microenvironment of neurons, such as leukocyte chemotaxis and Schwann cell migration [24–26]. Concurrently, the 1519 downregulated DEGs are mainly referred to the Cellular Components (presynapse, dendrite, axon, mitochondrial envelope, transsynaptic signaling, and endosomal part) and Biological Processes (transsynaptic signaling, synaptic signaling, chemical synaptic transmission, and anterograde transsynaptic signaling), as well as the pathway of dopaminergic synapse, which may indicate that the downregulated DEGs will negatively affect the synaptic structure and modulate the synaptic transmission [27–29]. Hub modules in the PPI network mainly referred to the translation and transcription, which will negatively impact the protein function.

Of the eleven Hub genes, 6 are upregulated in PT comparing with CT. C3-encoded protein plays a key role in the complement activation and neuroactive ligand-receptor interaction after its posttranslational modification [30]. Besides, significantly increased in the cortical brain of patients with refractory epilepsy comparing with nonepileptic lesions [31], C3 was also demonstrated significantly dysregulated in mesial temporal lobe epilepsies [32]. APLNR encodes a member of the G protein-coupled receptor gene family, and ANXA2 encodes a member of the annexin family. In addition to their role in regulating tumor progression [33, 34], APLNR is mainly involved in the neuroactive ligand-receptor interaction and vascular development [35, 36], while ANXA2 mainly participates in the modulation of depressive behavior and signal transduction pathways [37, 38]. CD44 encodes a cell-surface glycoprotein and affects the cell-cell interactions, cell adhesion, and migration, leading to a low survival in high-grade neuroblastoma, as well as synaptic remodeling and epileptogenesis [39–41]. GLU encodes a secreted chaperone and affects cell death, tumor progression, and neuroprotective role [42, 43]. CP encodes a metalloprotein, which has been considered neuroprotective in neurodegenerative diseases [44].

The 3 downregulated Hub genes in PT comparing with CT are MCHR2, HTR1E, and CTSG. MCHR2 encodes a receptor for melanin-concentrating hormone with G protein-coupled peptide receptor activity and involves in the pathway of neuroactive ligand-receptor interaction [35]. It has been also found that MCHR2 plays a role in the control of feeding and sleep-wake behavior [45, 46]. HTR1E encodes a protein with G protein-coupled receptor activity and neurotransmitter receptor activity [35]. CTSG encodes a member of the peptidase S1 protein family, which is found broadly expressed in acute myeloid leukemia and may participate in the connective tissue remodeling at the site of inflammation [47, 48].

The 2 downregulated Hub genes in PT comparing with NC are APP and GNG2. APP encodes a cell surface receptor and a precursor of several peptides. Besides participating in the pathogenesis of Alzheimer's disease due to the accumulation of amyloid-*β* peptide contributes [49], APP also plays a neuroprotective role in the synaptic transmission, plasticity, calcium signaling, and neuronal network activity [50]. It has been proved that APP can significantly promote synaptogenesis once the copper binds to the growth factor-like domain [51]. GNG2 encodes one of the gamma subunits of a guanine nucleotide-binding protein, which is a transmembrane signal transducer containing three subunits of alpha, beta, and gamma [52].

The 5 Hub genes, including C3, CD44, ANXA2, HTR1E, and APP, were also identified as the BottleNeck genes, that is, they are Hub-BottleNeck genes, indicating that they are much more important than the remaining Hub genes in the pathogenesis of epilepsy in PT.

## 5. Conclusions

In the present study, unique transcriptional characteristics of PT were identified by comprehensive bioinformatics methods, making it possible to distinguish PT from CT and NC. In addition to the enriched GO terms and KEGG pathways, eleven Hub genes were screened out. Furthermore, five Hub-BottleNeck genes were also identified, suggesting that they may have great potential significance for patients with TSC-associated epilepsy in the next step of diagnosis and treatment. Although the risk of false-positive results is high due to the small sample size and lack of experimental verification, it still provides new sights for further study of this disease. Finally, further basic experimental research and clinical verification with larger sample size are still needed to confirm the findings in the present study.

## Figures and Tables

**Table 1:** The enrichment analysis of DEGs between PT and CT clustered in hub modules.

Category	Term	Description	Count	*P* value
CC	GO:0035578	Azurophil granule lumen	5	4.06*E*-09
KEGG	hsa04080	Neuroactive ligand-receptor interaction	6	2.40*E*-08
BP	GO:0006875	Cellular metal ion homeostasis	6	1.85*E*-06
BP	GO:0006855	Drug transmembrane transport	3	2.48*E*-05
BP	GO:0051235	Maintenance of location	3	1.49*E*-03

BP: Biological Processes; CC: Cellular Components; DEG: differentially expressed genes; GO: Gene Ontology; has: Homo sapiens; KEGG: Kyoto Encyclopedia of Genes and Genomes.

**Table 2:** The enrichment analysis of DEGs between PT and NC clustered in hub modules.

Category	Term	Description	Count	*P* value
BP	GO:0043687	Post-translational protein modification	35	7.91*E*-33
BP	GO:0070125	Mitochondrial translational elongation	18	3.78*E*-23
BP	GO:0006283	Transcription-coupled nucleotide-excision repair	12	1.21*E*-14
BP	GO:0007187	G protein-coupled receptor signaling pathway, coupled to cyclic nucleotide second messenger	16	2.23*E*-12
CC	GO:0030665	Clathrin-coated vesicle membrane	12	3.30*E*-12
BP	GO:0001736	Establishment of planar polarity	12	8.15*E*-12
KEGG	hsa04062	Chemokine signaling pathway	12	7.33*E*-10
MF	GO:0001664	G protein-coupled receptor binding	14	1.01*E*-09
MF	GO:0004842	Ubiquitin-protein transferase activity	15	6.70*E*-09
MF	GO:0008528	G protein-coupled peptide receptor activity	10	1.40*E*-08

BP: Biological Processes; CC: Cellular Components; DEG: differentially expressed genes; GO: Gene Ontology; has: Homo sapiens; KEGG: Kyoto Encyclopedia of Genes and Genomes; MF: Molecular Functions.

## Data Availability

The data used to support the findings of this study are included within the article.

## Supplementary Materials

Supplementary 1 Table S1. The top 10 nodes in the PPI network ranked by Degree, MCC, and BottleNeck method, respectively (PT VS CT). PPI: protein-protein interaction; MCC: Maximal Clique Centrality; PT: perituberal tissue; CT: cortical tuber; VS: versus.

Supplementary 2 Table S2. The top 10 nodes in the PPI network ranked by Degree, MCC, and BottleNeck method, respectively (PT VS NC). PPI: protein-protein interaction; MCC: Maximal Clique Centrality; PT: perituberal tissue; NC: normal cortex; VS: versus.

Supplementary 3 Figure S1. The boxplots of NUSE for the MAQC. NUSE: normalized unscaled standard errors; MAQC: MicroArray Quality Control.

Supplementary 4 Figure S2. The RNA degradation map for the MAQC. MAQC: MicroArray Quality Control.

Supplementary 5 Figure S3. The PPI network for the DEGs between PT and CT. PPI: protein-protein interaction; DEGs: differentially expressed genes; PT: perituberal tissue; CT: cortical tuber.

Supplementary 6 Figure S4. The PPI network for the DEGs between PT and NC. PPI: protein-protein interaction; DEGs: differentially expressed genes; PT: perituberal tissue; NC: normal cortex.

Supplementary 7 Figure S5. The top 100 GO terms of down-regulated DEGs between PT and NC. GO: Gene Ontology; DEGs: differentially expressed genes; PT: perituberal tissue; NC: normal cortex.
