# Supplementary material for: The Important Role of Perituberal Tissue in Epileptic Patients with Tuberous Sclerosis Complex by the Transcriptome Analysis
Source: Biomed Res Int. 2020 Oct 15;2020:4980609. doi: 10.1155/2020/4980609 (PMC7585662; doi:10.1155/2020/4980609)
Supplement: Supplementary 1 — Table S1. The top 10 nodes in the PPI network ranked by Degree, MCC, and BottleNeck method, respectively (PT VS CT). PPI: protein-protein interaction; MCC: Maximal Clique Centrality; PT: perituberal tissue; CT: cortical tuber; VS: versus. [file 4980609.f1.docx]

| Degree | | | MCC | | | BottleNeck | | |
| --- | --- | --- | --- | --- | --- | --- | --- | --- |
| Rank | Name | Score | Rank | Name | Score | Rank | Name | Score |
| 1 | C3 | 13 | 1 | C3 | 46 | 1 | C3 | 44 |
| 2 | CD44 | 10 | 2 | MCHR2 | 30 | 2 | ANXA2 | 36 |
| 2 | ANXA2 | 10 | 3 | HTR1E | 26 | 3 | ITGB4 | 18 |
| 4 | MCHR2 | 7 | 4 | APLNR | 25 | 4 | CD81 | 13 |
| 5 | CLU | 6 | 5 | TAS2R39 | 24 | 5 | CD44 | 10 |
| 5 | PTGDS | 6 | 6 | ANXA2 | 15 | 6 | TNC | 9 |
| 5 | CTSG | 6 | 7 | CD44 | 13 | 7 | PTGDS | 6 |
| 5 | HTR1E | 6 | 8 | CLU | 10 | 8 | ACTA1 | 5 |
| 9 | FLNC | 5 | 8 | CP | 10 | 8 | SLC15A2 | 5 |
| 9 | CP | 5 | 8 | CTSG | 10 | 8 | CNGB1 | 5 |
| 9 | HLA-E | 5 |  |  |  | 8 | HTR1E | 5 |
| 9 | CD81 | 5 |  |  |  |  |  |  |
| 9 | APLNR | 5 |  |  |  |  |  |  |

**Table S1**. The top 10 nodes in the PPI network ranked by Degree, MCC and BottleNeck method, respectively (PT VS CT).

**Abbreviations:** PPI: protein-protein interaction, MCC: Maximal Clique Centrality, PT: perituberal tissue, CT: cortical tuber.
