# Supplementary material for: The Important Role of Perituberal Tissue in Epileptic Patients with Tuberous Sclerosis Complex by the Transcriptome Analysis
Source: Biomed Res Int. 2020 Oct 15;2020:4980609. doi: 10.1155/2020/4980609 (PMC7585662; doi:10.1155/2020/4980609)
Supplement: Supplementary 2 — Table S2. The top 10 nodes in the PPI network ranked by Degree, MCC, and BottleNeck method, respectively (PT VS NC). PPI: protein-protein interaction; MCC: Maximal Clique Centrality; PT: perituberal tissue; NC: normal cortex; VS: versus. [file 4980609.f2.docx]

| Degree | | | MCC | | | BottleNeck | | |
| --- | --- | --- | --- | --- | --- | --- | --- | --- |
| Rank | Name | Score | Rank | Name | Score | Rank | Name | Score |
| 1 | APP | 111 | 1 | CHRM2 | 5.11E+19 | 1 | APP | 40 |
| 2 | IL6 | 84 | 2 | APP | 5.11E+19 | 2 | ACTB | 25 |
| 3 | ACTB | 76 | 3 | GNG2 | 5.11E+19 | 3 | IL6 | 24 |
| 4 | PPP2R1A | 69 | 4 | LPAR3 | 5.11E+19 | 4 | CCT2 | 21 |
| 5 | POLR2F | 64 | 4 | GPR17 | 5.11E+19 | 5 | PPP2R1A | 18 |
| 6 | CCT2 | 61 | 6 | CXCL8 | 5.11E+19 | 5 | FAIM2 | 18 |
| 7 | GNG2 | 59 | 7 | ADCY6 | 5.11E+19 | 7 | YWHAE | 16 |
| 7 | HSP90AB1 | 59 | 7 | ADCY3 | 5.11E+19 | 8 | PRPF19 | 15 |
| 9 | POLR2E | 57 | 9 | GNAI2 | 5.11E+19 | 9 | PRKACA | 14 |
| 9 | MAPK14 | 57 | 10 | CXCR4 | 5.11E+19 | 9 | STAT1 | 14 |

**Table S2**. The top 10 nodes in the PPI network ranked by Degree, MCC and BottleNeck method, respectively (PT VS NC).

**Abbreviations:** PPI: protein-protein interaction, MCC: Maximal Clique Centrality, PT: perituberal tissue, NC: normal cortex.
