# Supplementary figures and images for: The Important Role of Perituberal Tissue in Epileptic Patients with Tuberous Sclerosis Complex by the Transcriptome Analysis
Source: Biomed Res Int. 2020 Oct 15;2020:4980609. doi: 10.1155/2020/4980609 (PMC7585662; doi:10.1155/2020/4980609)

# NUSE

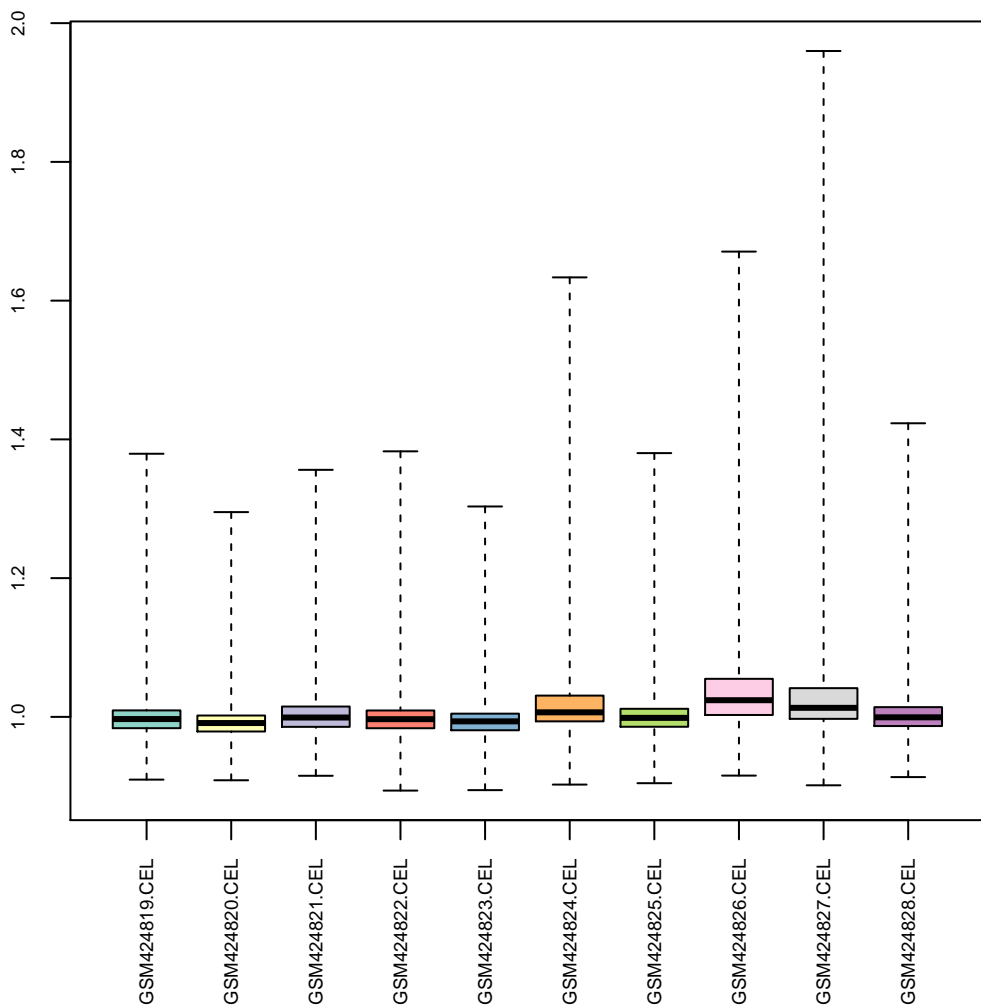

Supplement: Supplementary 3 — Figure S1. The boxplots of NUSE for the MAQC. NUSE: normalized unscaled standard errors; MAQC: MicroArray Quality Control. [file 4980609.f3.pdf]

# RNA degradation plot

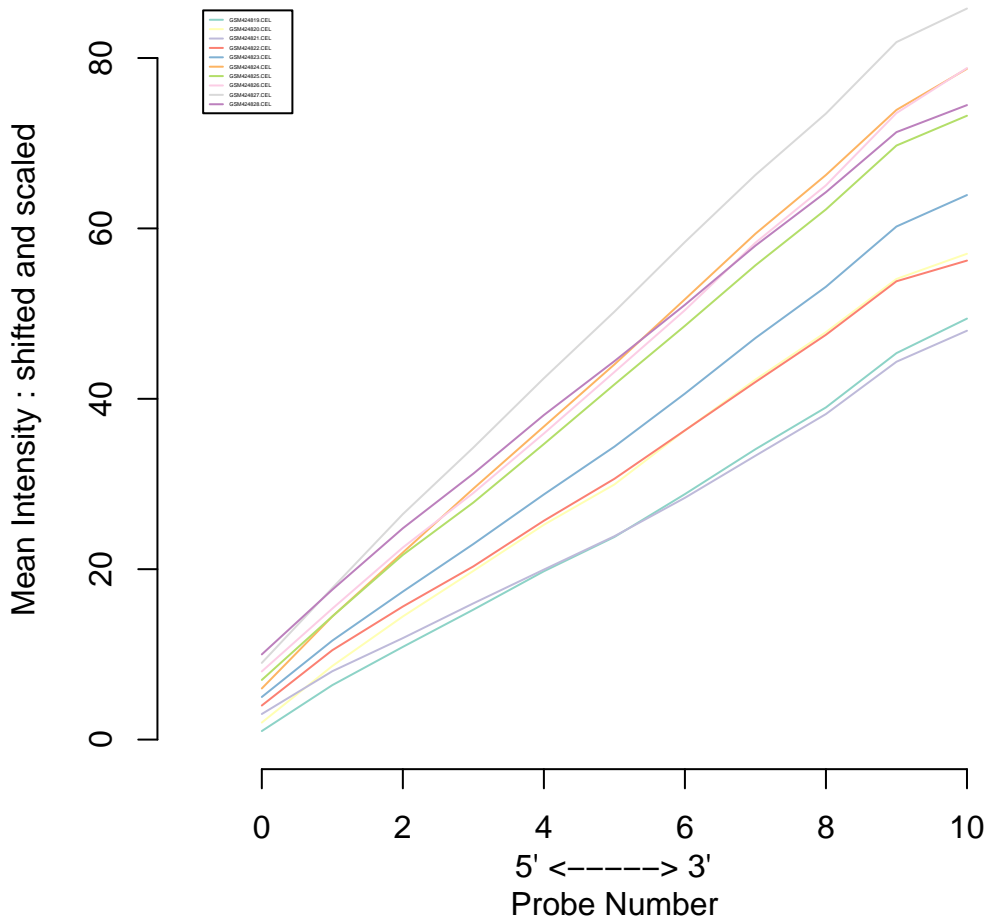

Supplement: Supplementary 4 — Figure S2. The RNA degradation map for the MAQC. MAQC: MicroArray Quality Control. [file 4980609.f4.pdf]

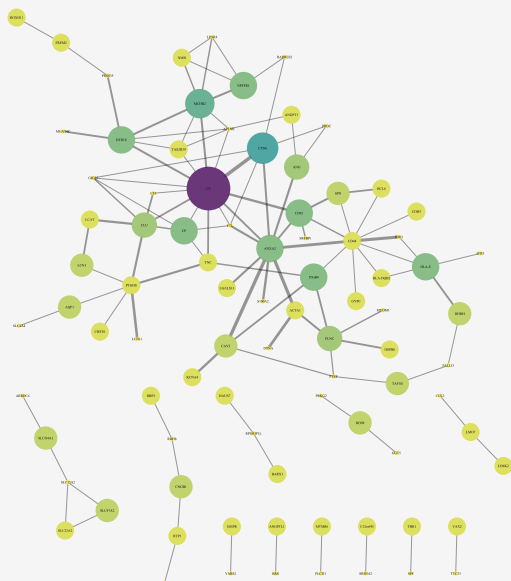

Supplement: Supplementary 5 — Figure S3. The PPI network for the DEGs between PT and CT. PPI: protein-protein interaction; DEGs: differentially expressed genes; PT: perituberal tissue; CT: cortical tuber. [file 4980609.f5.pdf]

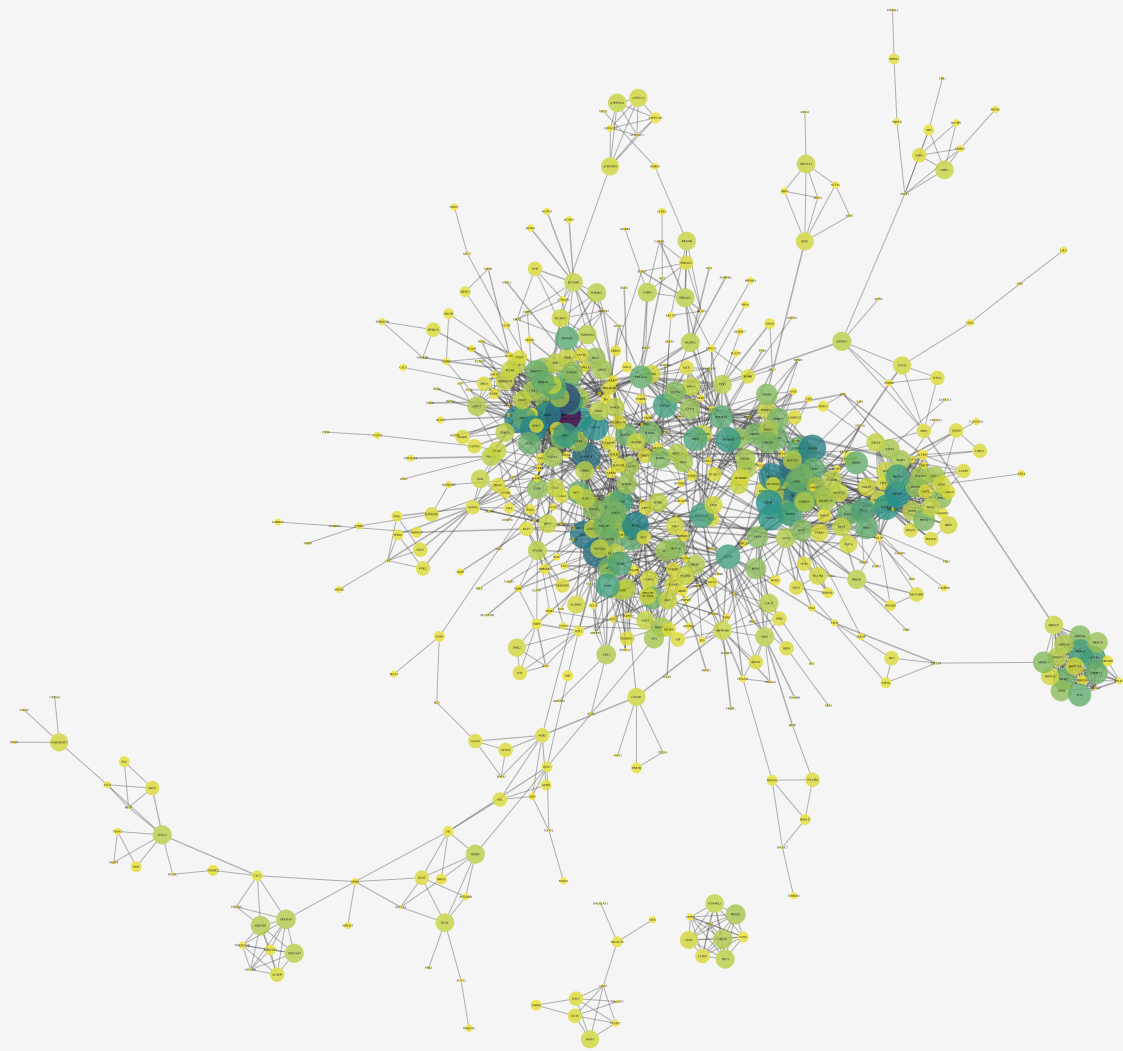

Supplement: Supplementary 6 — Figure S4. The PPI network for the DEGs between PT and NC. PPI: protein-protein interaction; DEGs: differentially expressed genes; PT: perituberal tissue; NC: normal cortex. [file 4980609.f6.pdf]

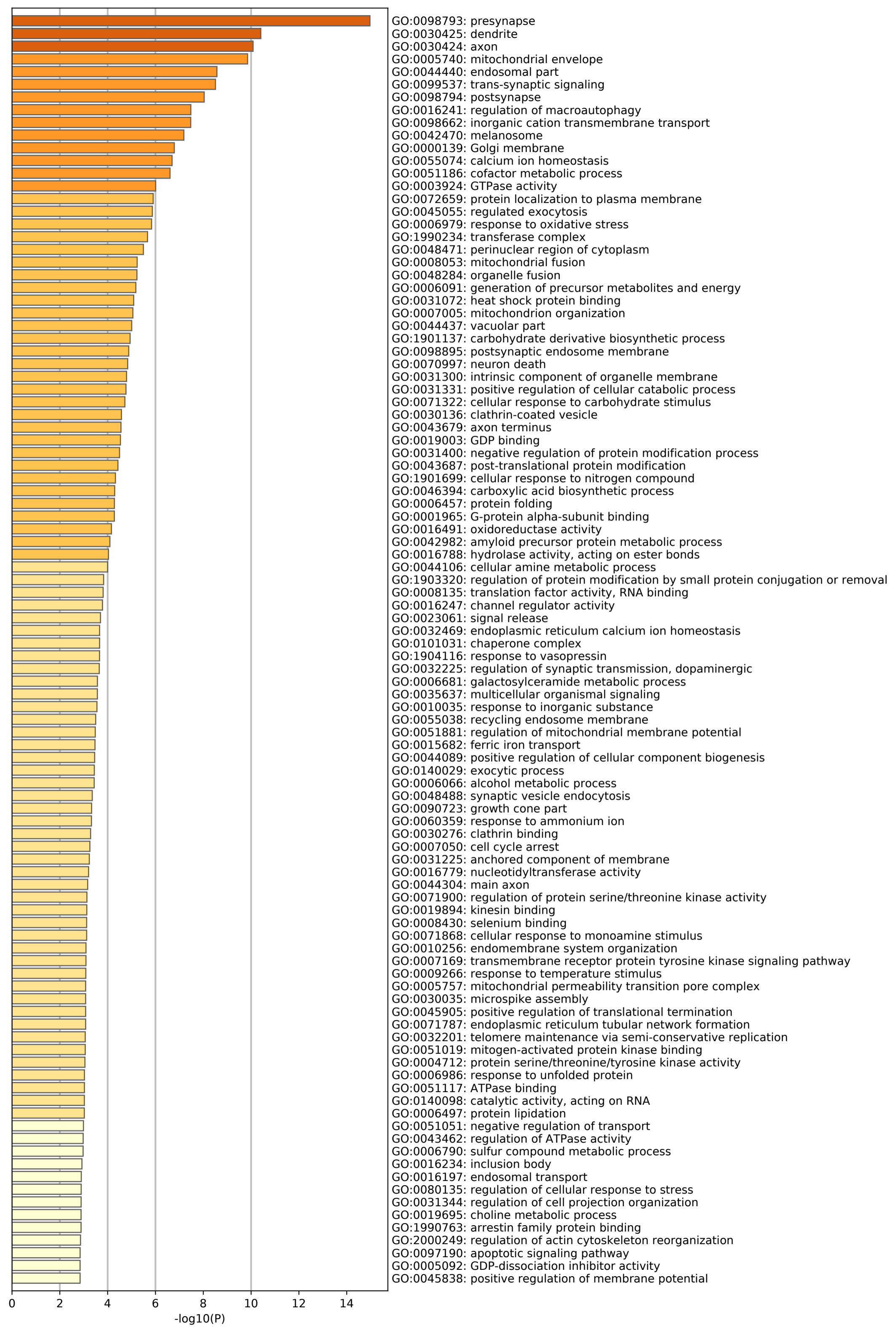

Supplement: Supplementary 7 — Figure S5. The top 100 GO terms of down-regulated DEGs between PT and NC. GO: Gene Ontology; DEGs: differentially expressed genes; PT: perituberal tissue; NC: normal cortex. [file 4980609.f7.pdf]
